# Prevalence of Metabolic Syndrome and Insulin Resistance in a Sample of Adult ADHD Outpatients

**DOI:** 10.3389/fpsyt.2022.891479

**Published:** 2022-06-21

**Authors:** Giulia di Girolamo, Irene Francesca Bracco, Alberto Portigliatti Pomeri, Soraya Puglisi, Francesco Oliva

**Affiliations:** ^1^Department of Neurosciences “Rita Levi Montalcini,” University of Turin, Turin, Italy; ^2^Department of Clinical and Biological Sciences, University of Turin, Turin, Italy

**Keywords:** metabolic syndrome, ADHD, insulin resistance, adult, obesity, BMI, age, hypertriglyceridemia

## Abstract

**Background:**

High prevalence of Metabolic Syndrome (MS) was found in patients with schizophrenia and bipolar disorders. Insulin Resistance (IR) seems to mediate MS role in developing cardiometabolic consequences.

**Aims:**

To investigate the prevalence of MS, and the role of MS components and IR surrogate indexes in determining MS in adult ADHD outpatients.

**Methods:**

In the present cross-sectional study, MS, defined according to the Expert Panel on Detection, Evaluation and Treatment of High Blood Cholesterol in Adults (ATP III), and IR surrogate indexes were assessed on a consecutive sample of adult ADHD outpatients. Logistic regression analysis was performed to evaluate the effect of each ATP III component and IR surrogate index in determining MS.

**Results:**

Seventeen out of 158 patients (10.8%, 95%CI = 0.064/0.167) fulfilled the ATP-III criteria for MS. A comprehensive comparison with prevalence in the reference population was hindered by the lack of patients over 60 in the study sample, however under this age no significant differences were found. Among MS components, blood triglycerides level (OR = 1.02, 95%CI=1.01/1.03, *p* = 0.001) was the main predictor for MS, followed by diastolic blood pressure (OR = 1.08, 95%CI=1.01/1.16, *p* = 0.024) and waist circumference (OR = 1.06, 95%CI=1.01/1.13, *p* = 0.029). Lipid Accumulation Product (LAP, OR = 1.0006, 95%CI=1.0003/1.0009, *p* < 0.001) outperformed Triglyceride-Waist Circumference (TG-WC, OR=1.03, 95%CI=1.01/1.04, *p* < 0.001) in predicting MS.

**Conclusions:**

More attention should be paid not only to MS but also to each ATP III component of MS and LAP in ADHD patients both at first assessment and during follow-up process.

## Introduction

Metabolic Syndrome (MS) is a widespread cluster of cardiometabolic risk factors (1) with a great impact on global health, and high morbidity, mortality rate, and public costs (2–4).

The worldwide prevalence of MS is difficult to assess because of the deep variation in habits and mean age shown by different populations. Data about the U.S. population suggest a prevalence of almost 35% mostly driven by a high central obesity rate (5, 6). Consistently, a study conducted in Italy on patients referring to their general practitioner's office for miscellaneous complaints found a prevalence of almost 33%. Patients with MS were noticeably older (mean age = 61.5, SD = 9.3 years) than those who did not meet the criteria (mean age = 56.3, SD = 12.6 years) (7). Ten years before, a prevalence of 18% in men and 15% in women was detected in the Italian general population. Even then, age played an important role because the MS rate changed from 3%, in people between 18 and 25, to 25% in those over 70 (8). A recent epidemiological survey conducted on the European general population (9) has confirmed that the prevalence of MS steadily increases with age.

A comprehensive meta-analysis showed that MS is 58% more frequent in patients with any psychiatric disorder than in the general population (10). High prevalence of MS was detected especially in patients with schizophrenia (11), bipolar disorder (12, 13), and major depressive disorder (14), mostly due to unhealthy lifestyles (15, 16), sleep-wake rhythm disruption (1), and side effects of pharmacological treatments (11, 17, 18). Attention-Deficit/Hyperactivity Disorder (ADHD) is one of the conditions whose correlation with MS has recently been investigated.

ADHD is a pervasive neurodevelopmental disorder resulting in a persistent pattern of inattention, hyperactivity, and impulsivity severe enough to interfere with social, academic, and occupational functioning (19). The onset of ADHD is during childhood with an estimated worldwide prevalence of 3.4% (95%CI = 2.6/4.5) (20), but diagnostic criteria seem to persist in 57% of patients during adulthood when the estimated prevalence is 2.8% (IQR = 1.8–4.1%) (21). A meta-analysis on prospective longitudinal studies has suggested that approximately two-thirds of children with ADHD continue to suffer from impairing symptoms in adulthood (22). Therefore, ADHD is a lifespan persistent disorder that brings with it a higher risk of both psychiatric and physical comorbidities (23, 24). Some recent meta-analyses have reported higher rates of different medical conditions among patients with ADHD than controls, such as migraine [OR = 1.32, (25)], asthma [OR = 1.34, (26)], atopic eczema [OR = 1.32, (26)], coeliac disease [OR = 1.39, (27)].

To the best of our knowledge, only a recent European study has investigated the relationship between ADHD and MS, also considering the impact of comorbid affective disorders. No significant differences in the MS prevalence have been detected between ADHD with anxiety/depressive disorders (23.9%), ADHD without anxiety/depressive disorders (19.9%) and controls (19.0%, χ^2^[2] = 2.08, *p* = 0.384) (28).

As stated before, the definition and the actual prevalence of MS have recently been challenged by some authors (29, 30), however, every single MS diagnostic criterion is still recognized as an independent risk factor for cardiovascular disease (31, 32).

Moreover, though it is well known that Insulin Resistance (IR) plays a key role in the MS development (33, 34), MS and IR are mutually independent predictors of cardiovascular risk (35) and IR seems to be one of the most significant determinants of endothelial dysfunction in MS (36). The most reliable procedure to assess IR is the hyperinsulinemic-euglycemic clamp that is too cumbersome in terms of time and cost. Homeostasis Model Assessment-Insulin Resistance (HOMA-IR), instead, still requires insulinemia determination, which is not commonly included in the standard blood panel. However, different surrogate indexes of IR have been proposed and recently validated (37–39). Among these Triglyceride-Waist Circumference (TG-WC) (39) and Lipid Accumulation Product (LAP) (37) have shown to be the most powerful predictors of MS (38, 40). Since TG-WC and LAP have been recently validated, no studies investigated them in ADHD patients.

ADHD is associated with a significant risk for overweight and obesity at all ages (41–43) and, beyond the actual or not ADHD diagnosis, the presence of inattentive and hyperactive/impulsive symptoms during adolescence correlates with larger waist circumference in adulthood (44). ADHD has also been found to significantly associate with type 2 diabetes, which implies IR, and hypertension (45, 46). Furthermore, a Body Mass Index (BMI) over 25 Kg/m^2^, a diastolic blood pressure higher than 90 mmHg, and an increased level in Low-Density Lipoprotein (LDL) are more likely to be present in adults with ADHD (47). A partial explanation for the metabolic risk could be found in unhealthy dietary habits which are known to be associated with ADHD including late-night eating (a behavior associated with either ADHD and increased BMI) (48, 49) and keeping irregular intervals between meals (50). ADHD also showed a high comorbidity rate with mood disorders (21, 51), which, as reminded before, are associated with a higher rate of MS (13, 52, 53).

The aims of the present study were (a) to investigate the prevalence of MS in an Italian naturalistic sample of adult outpatients with ADHD using the National Cholesterol Education Program—Adult Treatment Panel III (ATP III) criteria and (b) to evaluate the role of the IR surrogate indexes and MS components in determining MS.

## Materials and Methods

The present cross-sectional study was conducted at the adult ADHD outpatient center of the San Luigi Gonzaga University Hospital (Orbassano, Turin, Italy), between January 2019 and January 2020, on a consecutive naturalistic sample of patients newly diagnosed with adult ADHD according to DSM-IV-TR criteria (age>18 years). The recruiting center is located within the University General Hospital and it is a tertiary referral service for the diagnosis and treatment of ADHD in adults, covering the northwestern region of Italy.

The invitation to participate in the study was accompanied by comprehensive information regarding its aims, methods, risks, and benefits. If accepted, the agreement was formalized by the patient signing a written informed consent form. A unique identification code was assigned to each patient in order to maintain data anonymity and patient confidentiality. All procedures involving human subjects/patients were approved by the Research Ethics Committee of the San Luigi Gonzaga University Hospital (Orbassano, Turin, Italy; Prot no. 16/2019) as a part of a wider observational study project. Therefore, the authors assert that all procedures contributing to this work comply with the ethical standards of the relevant national and institutional committees on human experimentation and with the Helsinki Declaration of 1975, as revised in 2008.

ADHD diagnosis was assessed by a comprehensive psychiatric clinical interview followed by the administration of Adult ADHD Self Reporting Scale (ASRS-1.1) screener for adult ADHD; it was then confirmed through the Diagnostic Interview for Adult ADHD (DIVA 2.0). The severity of ADHD symptoms was rated by using the Adult ADHD Investigation Rating Scale (AISRS).

At the moment of their enrollment, patients were not taking any medication for ADHD as they were diagnosed for the first time at the center. Conversely, any other current psychopharmacological treatments were collected according to their main drug class (i.e., mood stabilizers, antidepressants, antipsychotics).

The metabolic syndrome was diagnosed according to the ATP III criteria. Thus, a dichotomous variable (Yes/No) for each MS component was obtained, using ATP III thresholds (i.e., waist circumference >102 cm in men and >88 cm in women; triglycerides levels ≥125 mg/dL; blood pressure ≥130/85 mmHg; High-Density Lipoprotein Cholesterol levels–HDL-C <40 mg/dL in men and <50 mg/dL in women; and fasting glucose values ≥100 mg/dL) and gathering any available information about current lipid-lowering, glucose-lowering, and antihypertensive medications. All patients having at least three out of five components were considered to be affected by MS and thus they were assigned to the MS group. The remainder of the sample was considered as belonging to the no MS group. Each component was also collected as a continuous variable, thereby weight, height, waist circumference and blood pressure were measured. BMI, defined as the ratio of body weight in kilograms and height in square meters, was calculated. Waist circumference, measuring central adiposity, was taken at the point in the middle between ribs' inferior margin and the superior edge of the iliac crest, while patients were exhaling. Blood pressure was detected using a mercury sphygmomanometer before and after examination, then the mean values for both diastolic and systolic blood pressures were collected. Blood concentrations of each blood test required (i.e., triglycerides, HDL-C, and fasting glucose) were derived from the standard blood panel carried on at the first examination.

IR surrogate indexes were calculated according to the original published formulas, i.e., LAP = (WC-65)*TG for men and (WC-58)*TG for women (37); TG-WC = TG*WC (39).

### Statistical Analysis

All computations were performed using RStudio for MAC OS (Version 1.1.383, RStudio Inc., Boston, MA, USA).

A comparison between MS group and no MS group was performed: Pearson's χ2 test or Fisher's exact test was used for categorical variables, depending on the expected frequencies in each group, whereas the independent samples Student's t test or the Mann-Whitney's U test was adopted for continuous variables, depending on their distributions previously evaluated by the Shapiro-Wilk's test.

The relationship between MS and age was even appraised comparing the prevalence of MS of the study sample with that of a normative population [i.e., taken from the most recent report on the prevalence of MS in the European general population (9)], both stratified by age.

Multicollinearity between MS components and IR indexes was also assessed by calculating the square root of variance inflation factor (√VIF), indicating the degree to which the confidence interval for each variable regression parameter is expanded relative to a model with uncorrelated predictors. As a general rule, √VIF > 2 was considered as an indicator of a multicollinearity problem (54).

The role of MS components and IR surrogate indexes in determining MS was evaluated by logistic regression. A first model (i.e., COMP model) was built including age and all MS components (i.e., waist circumference, systolic and diastolic blood pressures, triglycerides, HDL-C, and fasting glucose). According to multicollinearity assessment, two further models were performed excluding collinear MS components: The second model (i.e., LAP model) including LAP and the third model (TG-WG model) was with TG-WC.

Finally, the fitting level of the first model was compared to that of the second and the third model by anova() function of the MASS package and through Nagelkerke's pseudo-R^2^ and Akaike Information Criterion (AIC) calculation.

Probability tests were considered bilateral with a type I error set at 5% (*p* = 0.05) but *p*-values resulting from multiple comparisons were adjusted using Sidak's correction to control for the family-wise error rate.

## Results

The recruited sample consisted of 158 patients, 112 of which were males (70.9% of the total) and 46 females (29.1%); the mean age was 25 (SD = 14.0) years. The majority of them were referred to the center from a psychiatrist they had consulted for different ailments (*n* = 53 patients, 33.5% of the sample) but few less decided to undergo an evaluation for ADHD on their own initiative (*n* = 50 patients, 31.6%). The remaining patients were sent to the center by their child neuropsychiatrist (*n* = 23, 14.6%), by any association of ADHD children's families (*n* = 10, 6.3%), by their general practitioner (*n* = 2, 1.3%) or by their addiction service (*n* = 2, 1.3%). Eighteen patients (10.8%) were referred to the center by other sources.

Seventeen patients (10.8%, 95%CI = 6.4/16.7%) fulfilled the ATP-III MS criteria for MS, meeting at least three out of five requirements; 37 (23.4%) had the waist circumference criterion, 59 (37.3%) had the hypertension criterion, 43 (27.2%) had the triglycerides criterion, 45 (28.5%) had the HDL criterion, and only 8 (5.1%) had the hyperglycemia criterion.

The prevalence rates of MS stratified by age were compared with those of a normative population [Vishram et al. (9); *N* = 69.094]. Although the overall rate of our sample is about half of that of normative population (ADHD MS = 10.8% vs. normative MS = 20%, χ^2^[1] = 7.881, *p* = 0.005), no differences were found under 60 years (19–39 years, ADHD MS = 9.8% vs. normative MS = 9%, χ^2^[1] = 0.035, *p* = 0.852; 40–49 years, ADHD MS = 12.5 vs. normative MS = 20.2, χ^2^[1] = 0.205, *p* = 0.650; 50–59 years, ADHD MS = 25% vs. normative MS = 23.9%, χ^2^[1] = 0.006, *p* = 0.940). A comparison for elderly patients (above 60 years of age) could not be performed because of their paucity in the study sample (two patients, nobody with MS vs. 8671 patients, 2561 with MS).

### Comparison Between Groups for Categorical and Continuous Variables

**Figure 1:**
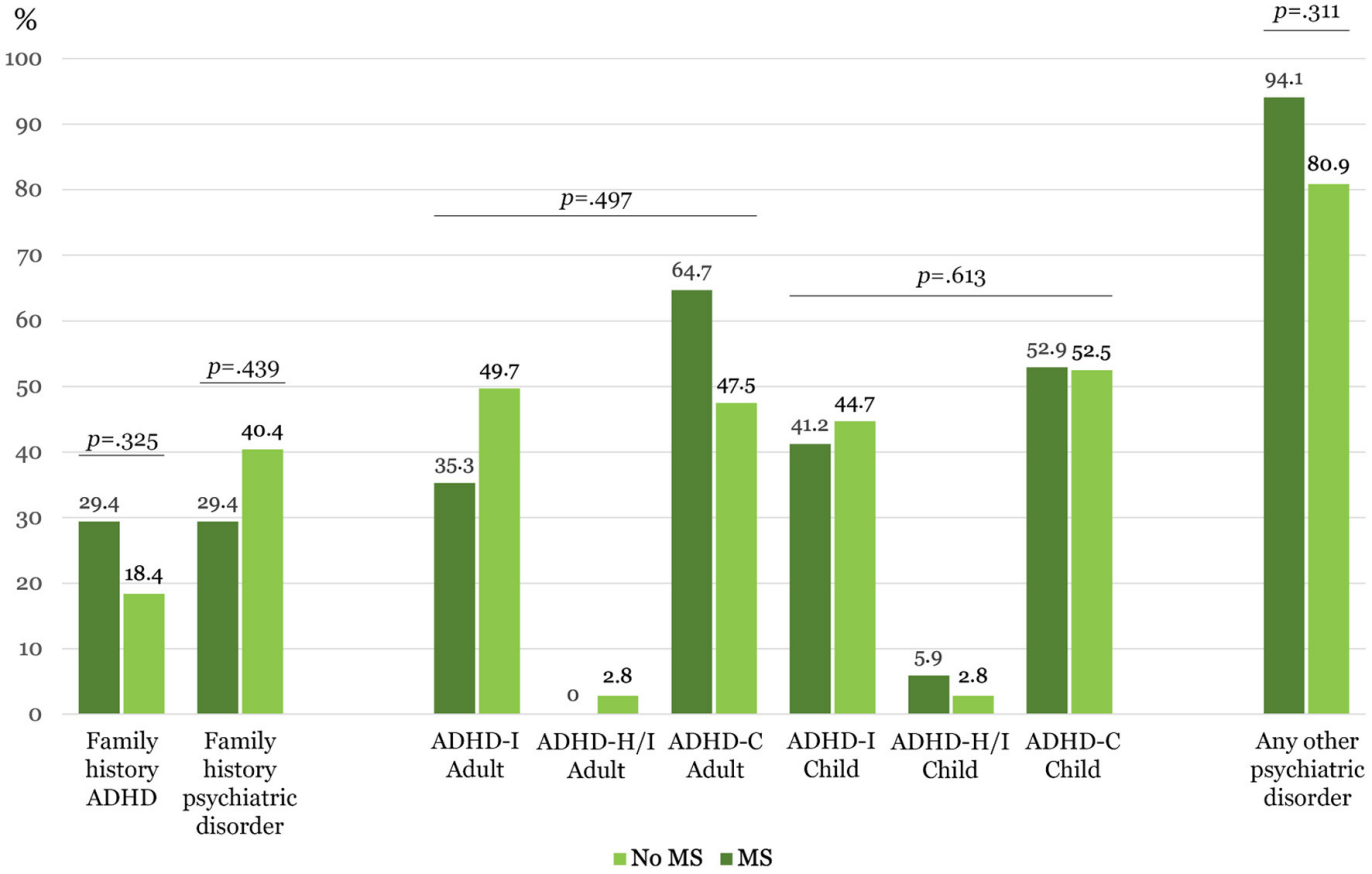
Clinical categorical variables (*N* = 158). ADHD-I, Predominantly inattentive presentation; ADHD-H/I, Predominantly hyperactive/impulsive presentation; ADHD-C, Combined presentation. ^*^Statistically significant after Sidak's adjustment, *p* ≤ 0.004.

No significant differences were found between MS and no MS groups regarding categorical variables including psychopharmacological treatments (Table 1, Figure 1).

**Table 1:** Socio-demographic and clinical categorical variables (*N* = 158).

	**Metabolic Syndrome**				
	**No** ***N*** **= 141 (89.2%)**	**Yes** ***N*** **= 17 (10.8%)**	**Total** ***N*** **= 158 (100%)**	**χ^2^/ Fisher**	**p**
**Sex** F M **Employment** Unemployed Student Employed **Education** Primary school Intermediate school High school Bachelor's degree Master's degree **Sleep** Regular Delayed onset Central insomnia Terminal insomnia Total insomnia Hypersomnia **Mood Stabilizers** Yes **Antidepressant drugs** Yes **Antipsychotic drugs** Yes	39 (27.7%) 102 (72.3%) 57 (40.4%) 40 (28.4%) 44 (31.2%) 1 (0.7%) 106 (75.2%) 18 (12.8%) 7 (5.0%) 9 (6.4%) 76 (53.9%) 48 (34.0%) 8 (5.7%) 4 (2.8%) 4 (2.8%) 1 (7%) 33 (23.4%) 31(22.0%) 19(13.5%)	7 (41.2%) 10 (58.8%) 6 (35.3%) 3 (17.6%) 8 (47.1%) 0 (0.0%) 9 (52.9%) 4 (23.5%) 3 (17.6%) 1 (5.9%) 8 (47.1%) 7 (41.2%) 1 (5.9%) 1 (5.9%) 0 (0.0%) 0 (0.0%) 4 (23.5%) 5 (29.4%) 3 (17.6%)	46 (29.1%) 112 (70.9%) 63 (39.9%) 43 (27.2%) 52 (32.9%) 1 (0.6%) 115 (72.8%) 22 (13.9%) 10 (6.3%) 10 (6.3%) 84 (53.2%) 55 (34.8%) 9 (5.7%) 5 (3.2%) 4 (2.5%) 1 (0.6%) 37 (23.4%) 36 (22.8%) 22 (13.9%)	1.343 1.899 6.838 2.425 <0.001 0.476 0.220	0.266 0.441 0.124 0.806 1.000 0.542 0.709

*Statistically significant after Sidak's adjustment, p ≤ 0.004.

As regards continuous variables (Table 2), diastolic blood pressure, waist circumference, triglycerides, LAP, TG-WC as well as weight and BMI were confirmed to be higher in the MS group than in the no MS group. Notably, no significant age difference was found between study groups.

**Table 2:** Socio-demographic and clinical continuous variables (*N* = 158).

	**Metabolic Syndrome**				
	**No** ***N*** **= 141 (89.2%)** **Mdn (IQR)/ M(SD)**	**Yes** ***N*** **= 17 (10.8%)** **Mdn (IQR)/ M(SD)**	**Total** ***N*** **= 158 (100%)** **Mdn (IQR)/ M(SD)**	**U/t (df)**	**p**
Age at assessment AISRS Weight, Kg Height, cm BMI, Kg/m^2^ Systolic blood pressure, mmHg Diastolic blood pressure, mmHg Waist circumference, cm Triglycerides, mg/dl HDL-C, mg/dl Fasting glucose, mg/dl	25.0 (11.0) 33.8 (8.54) 70.5 (18.5) 172.4 (9.61) 23.1 (4.80) 118.8 (17.0) 75.0 (14.0) 86.6 (20.0) 99.0 (74.0) 52.0 (17.0) 86.0 (15.0)	31.0 (12.0) 33.0 (8.0) 85.0 (18) 175.1 (10.5) 28.6 (5.20) 130.0 (20) 85.9 (10.0) 98.0 (24.0) 176.5 (67.0) 45.0 (13.0) 88.0 (15.0)	25.0 (12.75) 33.8 (8.0) 72.0 (20.0) 172.7 (9.72) 23.5 (4.73) 119.8 (20) 75.5 (15.0) 88.1 (22.0) 104.3 (93.0) 51.0 (16.0) 86.3 (15.0)	916.5^U^ 1337 (156)^U^ 264.5^U^ 0.88 (156)^t^ 278.0^U^ 840 (156)^U^ 14.34 (156)^t^ 3.78 (156)^t^ 449.0^U^ 2.83 (156)^t^ 1052.0^U^	0.114 0.437 0.002^*^ 0.392 0.003^*^ 0.044 <0.001^*^ <0.001^*^ <0.001^*^ 0.010 0.413
LAP	2255.4 (2561.8)	6565 (5100.7)	2441.2 (2941.0)	266.0^U^	<0.001^*^
TG-WC	308.9 (85.5)	375.2 (96.5)	315.6 (86.8)	451.0^U^	<0.001^*^

*AISRS, Adult ADHD Investigator Symptom Rating Scale; BMI, Body Mass Index; HDL-C, High-Density Lipoprotein Cholesterol; LAP, Lipid Accumulation Product; TG-WC, Triglycerides-Waist Circumference*.

U *, Mann-Whitney's U test*.

t *, Independent t-test*.

* *Statistically significant after Sidak's adjustment, p < 0.005*.

### Evaluation of the Role of Each MS Component and Each IR Surrogate Index in Determining MS

Multicollinearity was confirmed for both IR surrogate indexes (TG-WC, √VIF = 28.54; LAP, √VIF = 5.40), triglyceride (√VIF = 4.86), and waist circumference (√VIF = 23.33). Triglycerides and waist circumference were confirmed to be strongly correlated with IR surrogate indexes but not with each other. A significant correlation was detected also between IR surrogate indexes (Table 3).

**Table 3:** Correlation matrix of age, MS components and IR surrogate indexes.

	**Age**	**SBP**	**DBP**	**WC**	**TG**	**HDL–C**	**FG**	**LAP**	**TG–WC**
Age	1								
SBP	0.181	1							
DBP	0.162	0.208	1						
WC	0.092	0.118	0.143	1					
TG	0.044	0.092	0.073	0.128	1				
HDL–C	−0.023	−0.009	−0.116	−0.003	−0.040	1			
FG	−0.017	0.071	0.037	0.044	0.068	0.076	1		
LAP	0.112	0.147	0.154	0.596^*^	0.514^*^	−0.042	0.074	1	
TG–WC	0.095	0.141	0.150	0.772^*^	0.351^*^	−0.015	0.111	0.785^*^	1

*SBP, Systolic blood pressure; DBP, Diastolic blood pressure; WC, Waist circumference; TG, Triglycerides; HDL–C, High–Density Lipoprotein Cholesterol; FG, Fasting glucose; LAP, Lipid Accumulation Product; TG–WC, Triglycerides–Waist Circumference*.

* *Statistically significant after Sidak's adjustment, p < 0.006*.

The COMP model, including age and MS components, significantly explained the variation of MS (Nagelkerke's pseudo-R^2^ = 0.518, χ^2^[7] = 46.6, *p* < 0.001, AIC = 77.15). The main predictor was triglycerides blood level (OR = 1.02, 95%CI = 1.01/1.04, z = 3.27, *p* = 0.001), followed by diastolic blood pressure (OR = 1.08, 95%CI = 1.01/1.17, z = 2.24, *p* = 0.024), waist circumference (OR = 1.06, 95%CI = 1.01/1.13, z = 2.18, *p* = 0.029) and, with a weak protective effect, the HDL-C (OR = 0.93, 95%CI = 0.87/0.99, z = −2.05, *p* = 0.040).

Both the LAP and TG-WC model significantly explained the variation of MS (LAP model, Nagelkerke's pseudo-R^2^ = 0.546, χ^2^[6] = 49.7, *p* < 0.001, AIC = 72.18; TG-WC model, Nagelkerke's pseudo-R^2^ = 45.6, χ^2^[6] = 40.4, *p* < 0.001, AIC = 81.45). Nevertheless, the COMP model better fitted than the TG-WC model (deviance = −6.31, df = 1, *p* = 0.012) whereas it did not outperform the LAP model (deviance = 2.97, df = 1, *p* = 0.915). As triglycerides and waist circumference in the COMP model, both TG-WC and LAP confirmed to be significant predictors of MS in its relevant model (LAP, OR = 1.0006, 95%CI = 1.0003/1.0009, z = 4.14, *p* < 0.001; TG-WC, OR = 1.03, 95%CI = 1.01/1.04, z = 3.60, *p* < 0.001).

## Discussion

The overall prevalence of MS in our sample of adult ADHD outpatients fell into the range of the Italian general population (from 3% to 30%) (7, 8). This consistency with the general population still persisted when MS prevalence was analyzed in function of age. As already mentioned above, MS as well as any other cardiovascular risk factor tends to increase with age (55). According to the comparison between our ADHD sample and the most recent available normative data (9), no differences were found in age ranges between 18 to 60 but, above this age, the rate of MS in the general population continues to rise while in our sample it drops. This was easily explained by the young age of the study sample due to the paucity of patients above 60. As most of the patients are referred to the outpatient services from a psychiatrist or on their own, it can be assumed that older ADHD patients are less likely to be either recognized by doctors or sensitive to their own symptoms than younger adult patients.

Only over recent years, some authors have remarked the importance of underdiagnosis and untreated ADHD consequences in old adults and elderly people (56–59). A recent epidemiological study conducted on a Swedish national register (56) has shown that ADHD persists in elderly taking along psychiatric and metabolic comorbidity (over nine and two-fold prevalence than subjects without ADHD, respectively). However, according to another European study, no significant difference in lifestyle emerged when elderly ADHD patients were compared to same-age non-ADHD subjects (59). Therefore, more attention should be paid to this age group, which, by itself, seems to be at higher risk for cardiovascular complications. Since ADHD usually has a high prevalence of family history (60), its diagnosis may pose an indication to screen patients' parents for the same disorder to increase dysfunctional ADHD symptoms detection in elderly people.

The comparison with the prevalence of MS in other psychiatric disorders is hindered by the heterogeneity of different populations. Again, age plays a crucial role in determining different MS rates since older populations showed higher prevalence than our sample. In a recent systematic review and meta-analysis, the prevalence of MS was 33.4%, 31.7%, and 31.3% among patients diagnosed with schizophrenia, bipolar disorder and major depressive disorder, respectively (10), but the mean age of the overall population was 41.4 years (ranging from 22 to 73 years). An earlier review also found a bidirectional association between depression and MS but again the final sample age ranged from 20 to 91 years (14). In 2017, Moreira et colleagues carried out a controlled cross-sectional study among young patients (mean age = 25.81 ± 2.17) either with bipolar or major depressive disorder, experiencing a current depressive episode. The prevalence of MS (bipolar disorder = 46.9% and major depressive disorder = 35.1% vs. controls = 22.1%) was not significantly higher than that of healthy controls (12). Despite the similar mean age, the prevalence rate was higher than that detected in our ADHD sample, four-folds for bipolar, three-folds for depressive and even double for controls. Although this study focused on a young population, it considered only patients in the acute phase, thus limiting comparability with other studies. Moreover, differences in settings (acute stage of illness vs. outpatient assessment), regional factors, and pharmacological treatments can be held to explain this gap, especially with healthy controls.

The Italian clinical population was also investigated for the presence of MS (61, 62). Particularly, among bipolar patients, the role of age was confirmed. The prevalence of MS indeed increased from 9.1% under 30 years to 41.8% over 60 years, however, the sample mean age was 50.9 (SD = 15.5) years, way higher than that of the present study (62). Nevertheless, focusing on the prevalence of young patients only, our findings seem to be consistent with that reported by Salvi and colleagues (13) in their sample of Italian patients with bipolar disorder.

Fewer studies have focused on the relationship between OCD and metabolic complications (62, 63), but, in these patients, the main factor involved in increasing the risk of MS appeared to be the lifetime exposure to antipsychotic medications rather than other clinical or socio-demographic features (62).

Although a large proportion of our sample presented with at least one psychiatric comorbidity (82.3%), these conditions did not differ between the MS and no MS groups. This could once again be explained by the young age of the study sample which also implies a short duration of illness for comorbid conditions and thus a shorter exposition to treatments. As a matter of fact, the MS and the no MS group did not have significant differences in terms of ongoing medications. Furthermore, given the non-random allocation to treatments and the different metabolic risk profiles of each antipsychotic, it is expected that patients were receiving the best drug for their overall health status. This may lead to a confounding by indication bias.

Aside from strict metabolic criteria, BMI and weight significantly differed between groups, showing higher values in patients diagnosed with MS. These results are consistent with previous literature not investigating the presence of MS as a whole, but focusing on single metabolic risk factors (45, 47) and even on obesity (41–44). Nonetheless, the logistic regression analysis suggested that triglyceridemia was the main predictor of MS in our sample, as a rise of 1 mg/dl in blood triglycerides levels increases the MS risk of 0.2 points, which means that an increase of 50 mg/dl makes the patient positive for MS. Since blood triglycerides levels depend on dietary habits (e.g., calories intake and alcohol assumption) and weight (64), it is possible that the main driver for MS, in young adult ADHD patients, could be an unhealthy lifestyle rather than a reciprocal predisposition common to both the metabolic and the psychiatric condition (14). Because there is no evidence for more eating and drinking bad habits among elderly patients with ADHD (59), it can be assumed that triglycerides could be lower and this possibly could influence the prevalence rate of MS in this age group. Hence, further studies should be conducted including also older patients to estimate the actual rate of MS and of its main predictors.

As regards IR surrogate indexes, LAP seemed to perform better than TC-WC in predicting the risk for MS in our adult outpatient ADHD sample.

Although the relationship between ADHD and type 2 diabetes was investigated by Landau and Pinhas-Hamiel (46) this is the first study expressively reporting on OR indexes in adult ADHD patients.

To reduce atherosclerotic cardiovascular disease risk in MS, lifestyle interventions are the first-choice, regardless of age (65). Non-pharmacological approaches are recommended to all patients, including quit smoking, follow healthy diet and practice physical activity (66, 67). Pharmacological strategies such as statins and antihypertensive medications should be reserved to patients with specific risk profile based on previous history of cardiovascular disease, and Framingham-based 10-year risk score (65, 68). Fibrate could be an option if triglycerides ≥ 500 mg/dL or if non-HDL cholesterol remains elevated after maximum tolerated high intensity statin and ezetimibe (68). Recent data seem to support that also red yeast rice preparations can reduce mortality and major adverse cardiovascular events, with improvement in lipidic and glycemic serum levels, and blood pressure (69). Metformin has shown efficacy in decreasing weight/BMI and reducing progression toward diabetes mellitus (70), but data on cardiovascular risk reduction are not this straightforward. Although improvements have been reported with bariatric surgery (71), an accurate risk-benefit analysis should be performed before exposing the patient to surgical risks.

Target mechanisms for new medications include increasing insulin sensitivity, reducing appetite, decreasing pro-inflammatory cytokines release by adipose tissue, and counteracting both endothelial dysfunction and thrombogenic effects of MS (68).

Among the limitations of the present study, the lack of patients over 60 years of age is certainly the most important because it has not allowed us to properly evaluate the relationship between ADHD and MS, given the central role played by age, reducing also the possibility of confronting and generalizing our results. Secondly, in the present study, data concerning lifestyle habits (i.e., diet, physical activity, smoking, alcohol) and details on the exposition to medications (i.e. second-generation antipsychotic), which might have contributed to the development of MS, were not collected. Another limitation was the absence of a control group, which was addressed through a comparison of the age-stratified prevalence rate of MS with those of a European population. However, through this artifice we could reach reliable findings, at least for under 60 age groups. Finally, although the cross-sectional design limited the inferences on temporal relationships among variables, we could anyway perform a logistic regression to assess the contribution of each criterion in defining MS in our sample. Further, possibly perspective, studies are needed to better clarify the extent of cardiovascular risk in terms of metabolic factors among adult patients with ADHD.

In conclusion, we recommend clinicians to thoroughly screen ADHD patients for each MS component and to repeat the evaluation during the follow-up, paying particular attention to blood triglyceride concentration, inasmuch as they seem to predict the risk of a full MS.

## Data Availability Statement

The original contributions presented in the study are included in the article/supplementary files, further inquiries can be directed to the corresponding author.

## Ethics Statement

The studies involving human participants were reviewed and approved by Research Ethics Committee of the San Luigi Gonzaga University Hospital (Orbassano, Turin, Italy; Prot no. 16/2019). The patients/participants provided their written informed consent to participate in this study.

## Author Contributions

FO and GG conceived the study and drafted the manuscript together with IB who also collected the data. FO performed the statistical analysis. AP participated in the initial design of the study. SP contributed to revising the manuscript. All authors read and approved the final manuscript.

## Conflict of Interest

The authors declare that the research was conducted in the absence of any commercial or financial relationships that could be construed as a potential conflict of interest.

## Publisher's Note

All claims expressed in this article are solely those of the authors and do not necessarily represent those of their affiliated organizations, or those of the publisher, the editors and the reviewers. Any product that may be evaluated in this article, or claim that may be made by its manufacturer, is not guaranteed or endorsed by the publisher.
